# The trochoblasts in the pilidium larva break an ancient spiralian constraint to enable continuous larval growth and maximally indirect development

**DOI:** 10.1186/s13227-017-0079-5

**Published:** 2017-10-25

**Authors:** George von Dassow, Svetlana A. Maslakova

**Affiliations:** Oregon Institute of Marine Biology, University of Oregon, P.O. Box 5389, Charleston, OR 97420 USA

**Keywords:** Nemertea, Pilidium, Spiral cleavage, Cell fate specification, Fate map, Maximally indirect development

## Abstract

**Background:**

Nemertean embryos undergo equal spiral cleavage, and prior fate-mapping studies showed that some also exhibit key aspects of spiralian lineage-based fate specification, including specification of the primary trochoblasts, which differentiate early as the core of the prototroch of the spiralian trochophore larva. Yet it remains unclear how the nemertean pilidium larva, a long-lived planktotroph that grows substantially as it builds a juvenile body from isolated rudiments, develops within the constraints of spiral cleavage.

**Results:**

We marked single cells in embryos of the pilidiophoran *Maculaura alaskensis* to show that primary, secondary, and accessory trochoblasts, cells that would make the prototroch in conventional spiralian trochophores (1q^2^, 1q^12^, and some descendants of 2q), fully account for the pilidium’s primary ciliary band, but without undergoing early cleavage arrest. Instead, the primary ciliary band consists of many small, albeit terminally differentiated, cells. The trochoblasts also give rise to niches of indefinitely proliferative cells (“axils”) that sustain continuous growth of the larval body, including new ciliated band. Several of the imaginal rudiments that form the juvenile body arise from the axils: in particular, we show that cephalic imaginal disks originate from 1a^2^ and 1b^12^ and that trunk imaginal disks likely originate from 2d.

**Conclusions:**

The pilidium exhibits a familiar relation between identified blastomeres and the primary ciliated band, but the manner in which these cells form this organ differs fundamentally from the way equivalent cells construct the trochophore’s prototroch. Also, the establishment, by some progeny of the putative trochoblasts, of indeterminate stem cell populations that give rise to juvenile rudiments, as opposed to an early cleavage arrest, implies a radical alteration in their developmental program. This transition may have been essential to the evolution of a maximally indirect developing larval form—the pilidium—among nemerteans.

**Electronic supplementary material:**

The online version of this article (doi:10.1186/s13227-017-0079-5) contains supplementary material, which is available to authorized users.

## Background

The nemertean pilidium larva epitomizes maximally indirect development in which the morphogenesis, anatomy, and function of the larva and adult have minimal overlap, and the transition between the two life-history stages is accomplished via a rapid and catastrophic metamorphosis to avoid a prolonged state of dysfunction [1–3]. Uniquely in pilidial development, the juvenile worm forms from a set of eight initially isolated rudiments, including the three pairs of imaginal disks (cephalic, trunk, and cerebral organ disks) and unpaired dorsal and proboscis rudiments [1]. After many weeks of development inside the pilidium, the juvenile suddenly emerges and devours its larval body in a matter of minutes, instantaneously converting a planktonic microalgal grazer (the pilidium) into a benthic predatory adult [1, 4, 5]. Most benthic marine invertebrates display some form of indirect development [6], but only in a few groups do the larva and adult reach such a high degree of segregation as in the pilidium. Pilidial body plan and development are unique among animals and appear to have evolved within one clade of nemerteans, the Pilidiophora, from a more direct developmental trajectory [7–10]. Our results reveal a specific modification of the ancestral developmental program that may have enabled the evolution of a novel larval body plan and maximally indirect development in this group.

Nemerteans, a phylum of ~ 1300 described species of marine worms [11], belong to one of the most diverse bilaterian lineages, the Spiralia (Lophotrochozoa) [e.g., 12–18]. The Spiralia, whose radiation predates Cambrian [19], includes over half of all extant animal phyla, and many members of this clade exhibit a highly conserved pattern of early development called spiral cleavage [20–22]. Nemertean early development conforms to the embryological stereotype that characterizes this supraphyletic lineage [23–26]. In typical spiralian development, the first two cell divisions are meridional and produce four blastomeres—the founders of the four embryonic quadrants, denoted A, B, C, and D. These founder cells undergo a series of synchronized divisions producing four sets of usually smaller cells toward the animal pole—the four quartets of micromeres. These early divisions occur at oblique angles with respect to the animal–vegetal axis of the embryo, and the alternation of the angle with each successive division results in the characteristic “spiral” arrangement of blastomeres in the early embryo which gives the group its name.

The predictability of cell position and birth order in spiralian embryos is expressed in a nomenclature identifying each cell by quartet and quadrant of origin, appended with an indicator that tracks divisions since quartet birth (e.g., 2b^12^ is two divisions removed from the second quartet in the B quadrant). A set of four cognate cells, one in each quadrant, may be collectively referred to as “q”, e.g., 1q or 2q^12^. Notably, it is not only the arrangement of cells in the embryo that is highly conserved across spiralian phyla, but also the cell fates. For example, the 4d cell in most spiralians that have been examined to date gives rise to all of the adult mesoderm, while the 2d cell produces most of the ectoderm of the adult trunk [27, but see 28]. First-quartet micromeres (1q) give rise to the larval/adult head, and in many spiralians, the vegetal daughters of the first-quartet micromeres—1q^2^—each divide once or twice, exit the cell cycle, and become multiciliated. These form the main portion of the prototroch, the primary ciliary band of the spiralian trochophore larva, found in annelids sensu lato (incl. echiurids, sipunculids) and mollusks [29, 30].

The prototroch is a distinct pre-oral equatorial ciliary band involved in larval swimming and sometimes feeding, and it appears to have evolved only once among spiralians [31]. The prototroch typically consists of 25–40 large multiciliated cells derived from specific founders, called trochoblasts. The prototroch is a quintessentially larval organ and is lost before or during metamorphosis. Aside from the centrally positioned large “primary trochoblasts” (derived from 1q^2^), additional “accessory trochoblasts” (derived from 1q^12^) and “secondary trochoblasts” (derived from 2q) contribute to the prototroch of a typical spiralian [29, 30]. The early and irrevocable commitment of the spiralian trochoblasts to a terminal fate reflects one solution to a convergence of elementary constraints on the cellular basis of animal development. Because the cilium requires the centriole to serve as basal body, ciliary motility and mitosis are, for animal cells, mutually exclusive [32–38]. For larvae to swim effectively using cilia, they must have many cilia. This can be achieved either by many cells with one cilium each, in which case cells retain the potential to divide by transiently resorbing the cilium [e.g., 36], or by a few cells bearing many cilia each. The latter alternative—of which the prototroch is one instance—allows an embryo to swim early in development while devoting only a fraction of cells to motility, but at the cost that once a cell (at least, an animal cell) commits to produce the many centrioles required to support multiple cilia, it can likely never divide normally again.

Although it appears that the ancestral nemertean larva was uniformly ciliated [7, 8, 10], a vestigial prototroch composed of large multiciliated cells derived from the classical spiralian lineage has been identified in a palaeonemertean *Carinoma* [39]. Although these cells do not form a differentially ciliated band, the similarity in fates of defined blastomeres unifies the development of these direct-developing nemerteans with the classic stereotype for trochophore-forming spiralians such as annelids and mollusks. The previously published fate map for the pilidium larva of *Cerebratulus lacteus* likewise establishes several correspondences with the spiralian plan, including fundamental regional divisions and formation of mesoderm by 4d [25]. However, although the pilidium possesses an extensive differentially ciliated band, this band is clearly not a conventional prototroch, as one would find in a limpet or chiton trochophore: the pilidial ciliary band consists of far more cells and grows throughout larval life [40]. Henry and Martindale [25] showed that the pilidial ciliary band is derived mostly from the same quartets as the prototroch of other spiralians (1q and 2q), with a small addition by the third-quartet cells, 3c and 3d, but did not specifically follow the fates of the putative trochoblasts. Here, we complete the fate map of the pilidial ciliated bands by determining the fates of 1q^12^, 1q^2^, 2q, and 3q in the pilidium of *Maculaura alaskensis.* Remarkably, the same cells that form the prototroch in classical spiralians also account for the entire primary ciliated band of the pilidium, but by means of extensive cell division rather than precocious cleavage arrest. Indeed, our current results show that the 1q^2^ and 1q^12^ cells, far from limiting their proliferation, are the founders of the axillary growth zones previously shown to account for the continued growth of the larval epidermis and to give rise to the imaginal disks [40]. This in turn suggests that a radical change in the behaviors of these specific cells played a key part in the origin of a novel larval body plan, the pilidium, and its maximally indirect development.

## Methods

### Animals and embryos

*Maculaura alaskensis,* formerly known as *Micrura alaskensis* [41], adults were collected by shovel from intertidal sand/mudflats around Charleston, Oregon, in June through September of 2011–2016. Eggs were obtained by dicing a section of a gravid female in microfiltered natural seawater (MFSW). Eggs released into natural seawater spontaneously undergo germinal vesicle breakdown and develop a meiotic spindle, but await fertilization to initiate development. Sperm were released by dicing a section of a gravid male, and eggs were inseminated by adding an amount of dilute sperm suspension sufficient that a few sperm could be observed around each egg. Egg batches with large numbers of defective, undersized, or non-maturing eggs were discarded.

### Caged fluorescein dextran

The succinimidyl ester of CMNB-caged fluorescein (Invitrogen) was mixed with four micrograms of 10 kD or 70 kD amine-reactive dextran (Invitrogen) in 0.5 mL of 0.1 M borate at pH 8.5 and reacted in the dark overnight at 33 °C. The mixture was passed over a Zeba column (Pierce) and then loaded into a 3000-MWCO spin concentrator. Three rounds of ~ sixfold buffer replacement to aspartate injection buffer (AIB: 100 mM K-Aspartate, 50 mM KCl, 10 mM HEPES, pH 7.2) yielded a 10 mg/ml stock, which was aliquoted and stored at − 80 °C.

### Microinjection

The egg of *M. alaskensis* is ~ 75 μ in diameter and possesses a loose jelly that extends as much as two radii beyond the egg surface. The jelly prevents eggs from packing closely together and from approaching the dish surface. To deprive them of their jelly, fertilized eggs at the time of polar body emission were transferred to MFSW in glass dishes coated with bovine serum albumin (BSA, 1% in MFSW) and repeatedly sheared by passage through a drawn capillary with inner diameter ~ 150 μ (about twice egg diameter). Most batches of eggs packed closely after 5–10 passes, without losing the polar bodies. For injection, eggs were then transferred to MFSW in coverslip-bottomed dishes (MatTek) that had been swabbed with EtOH, rinsed in deionized water, and air-dried. Dishes so treated are just sticky enough that eggs can be injected, but can be released by slight shaking thereafter.

Needles were pulled from standard glass capillaries (Sutter) on a Sutter P97 and backfilled with a mixture of caged fluorescein and rhodamine dextran in AIB. We experimented with several variants, but ~ 5 mg/ml caged-FITC 70 kD dextran + 0.5 mg/ml TRITC 10 kD dextran (Invitrogen) gave us the best balance of brightness for both sorting by eye and scanning on the confocal. 70 kD dextrans are mostly excluded from the nucleus, whereas 10 kD dextrans enter it passively; this trait proved useful, despite the much lower brightness of the 70 kD dextran, because it reveals the number of nuclei in both labeled and unlabeled domains.

Zygotes were injected on a Leica DMIL inverted compound microscope using an MN-4/MMO-202D micromanipulator with a hydraulic joystick (Narishige) and PMI-200 pressure injector (Dagan). Needles were opened by breaking to a < 5-μ tip against a chip of glass placed into the injection dish. Fertilized eggs of *M. alaskensis* usually resist intrusion but not deformation and can be readily skewered into a torus without breaching the membrane. Therefore, the needle was pressed into the cell surface slightly; then, a light tap on the microscope frame was usually sufficient to break through. Eggs were pressure-injected with < 2% cytoplasmic volume, as judged by the diameter of the puff within the egg cytoplasm. Larger volumes caused embryos to bleb from the injection site, if not to lyse outright. Because of the obvious possibility that egg damage might alter cell fate specification, we adjusted injection volumes to the point that it was not possible to tell the vast majority of injected from uninjected eggs without fluorescence.

All data shown here come from experiments in which zygotes were injected. In some preliminary experiments, we injected 1 of 2, 1 of 4, or 1 of 8 cells and then uncaged a subset of descendant cells. We were not able to consistently inject 1 of 16 without damaging the target cell. Injecting 1 of 4 cells with the dextran mixture has the virtue that it minimizes spillover of the uncaging due to light scattering (discussed below). However, our data from these attempts showed a pronounced bias against the D quadrant, even though the fates revealed in other quadrants corresponded well with the results from injecting zygotes. We therefore abandoned injection of individual blastomeres.

Most batches of *M. alaskensis* eggs and embryos develop apparently normally [1] at steady temperatures between 10 and 20 °C. Therefore, we limited time spent on the uncooled injection scope to less than 15 min. This time is usually sufficient to inject 100–200 eggs. Before and after microinjection, eggs were cultured at 11 or 14 °C on Peltier-driven cold plates (Torrey Pines Scientific). Under this regime, we noticed no overt increase in developmental abnormalities, cleavage alterations, or delays between injected and uninjected embryos.

### Cell marking

After injection and cleavage, embryo batches were sorted manually to eliminate uninjected, damaged, or delayed embryos. At or immediately before the 16-cell stage, injected embryos in MFSW were transferred into a coverslip-bottomed dish rendered nonstick by brief treatment with 1% BSA in MFSW. The well in the middle was cover-slipped to limit water movement, then the dish filled with MFSW and placed in a Peltier-driven cooling stage insert (Dagan) at 14 °C on the stage of an Olympus FluoView 1000 confocal microscope equipped with a SIM scanner. Embryos were loose in this chamber. Embryos were selected for normal cleavage pattern and based on their orientation with respect to the targeted cell. To avoid confusion, only one quartet was targeted per dish. Target cells were marked by uncaging fluorescein using 405 nm light. In most experiments, this means 1- to 3-s illumination using the SIM scanner in a 5- to 10-μ spot at 1–2% intensity and an intermediate dwell time (20 μs/pixel).

This cell-marking regime proved highly reliable once we had identified workable parameters. However, one factor in particular shaped our methods: the yolk of *M. alaskensis* appears to strongly scatter violet light. This means that despite great care to illuminate just one cell in the embryo, prolonged illumination uncages some dye in other, not necessarily adjacent, cells. If not monitored carefully, spillover confounds interpretation of marked domains, especially since cells in the 4-day-old pilidium larva have diverse sizes and shapes. We experimented with several marking methods, including using the main scanner on the confocal (at 50% power, with a similar spot size and dwell time to the SIM scanner), using a MicroPoint laser (Photonic Instruments) operating at 365 nm, and using a home-made pinhole pierced in aluminum foil, then centered in the field diaphragm of a conventional epifluorescence microscope (Olympus BX51). All of these were effective, but none cured the spillover due to scatter. The only way we found to avoid it was to limit the uncaging stimulus: as uncaging in the target area approaches saturation, the amount of off-target uncaging due to scatter increases relative to the amount due to direct illumination. In other words, we found that we could only achieve brighter marking at the cost of specificity; hence, in virtually all larvae we analyzed, faint versions of one or more off-target cells’ domains pollute the image. Individuals were only excluded from our scoring if the spillover was bad enough that relative brightness in a ratio image (see below) could not clearly distinguish target from off-target domains.

After marking, embryos were immediately released from underneath the coverslip, marked embryos were sorted from unmarked by hand using a dissecting microscope (Leica MZ10F) equipped with epifluorescence and then cultured in the dark at 14–16 °C.

### Larval imaging and image processing

For examination, small groups of live 3- to 5-day-old larvae were anesthetized for 2–5 min in 50% MFSW:50% 0.35 M MgCl_2_ in a small petri dish, into which had been sprinkled a few grains of cetyl alcohol. This agent, which has no apparent short-term effects on larval behavior, greatly reduces the chances that MgCl_2_-relaxed larvae are destroyed by encounters with the air–water interface. Larvae were then transferred to a coverslip and stunned with sodium azide at ~ 0.2% final concentration. This dose, which would outright kill most ciliated invertebrate larvae, slows ciliary motion enough that a second coverslip, supported by vacuum grease ridges and pressed down until the larva is compressed to about 2/3 normal thickness, completely immobilizes the pilidium. Larvae can recover from this treatment if released into azide-free seawater within 15 min and apparently feed and grow thereafter; however, because we have not evaluated whether azide treatment has long-term consequences, all data shown here are from larvae so treated for the first time. These preps were made with two coverslips to enable larvae to be imaged from both sides at high resolution.

All imaging was conducted with an Olympus FluoView 1000 confocal system mounted on an Olympus IX81 inverted microscope equipped with 60 × 1.2 NA water-immersion lens, at 1× zoom and using 0.6 μ Z-steps. Because of the wide variation in cell aspect ratios within the pilidium—for example, “tile” cells of the epidermis are as much as 50 μ wide but only a micron or two thick, whereas ciliated band cells are much more compact—all data presented here are in the form of ratio images (fluorescein/rhodamine), to compensate for increased brightness due to cell thickness. To facilitate computation of accurate ratio images, detector sensitivity was adjusted to provide a small but positive background value in both fluorescence channels and to avoid saturation within marked domains. Ratios were computed using ImageJ as follows: (1) individual channels were median-filtered; (2) the fluorescein channel was multiplied to equalize background tissue fluorescence with the rhodamine channel, such that the ratio in an unmarked domain is close to 1.0; (3) true background (i.e., outside the larva) was raised to 100/4096 in both channels; (4) the ratio of fluorescein to rhodamine was computed, and (5) brightest-point projected. Resulting images were adjusted for black and white point only. In a few instances, to display adjacent marked and unmarked zones, the ratio image was log-transformed. False-color merges were prepared in Photoshop using “screen” compositing to preserve the luminance of the ratio image.

## Results

### Anatomy and major epidermal cell types of the pilidium larva

**Fig. 1:**
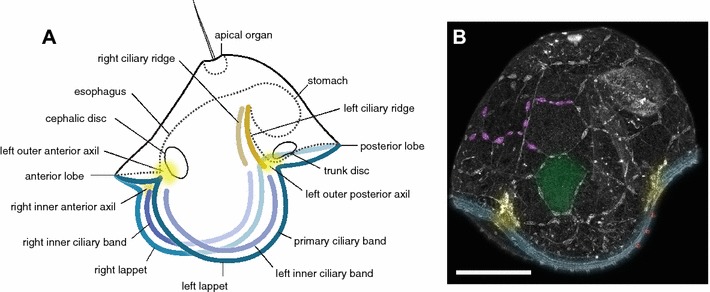
Anatomy and major epidermal cell types of the pilidium larva. **A** Diagram of a trunk-disk stage pilidium (~ 2 weeks old) illustrating major anatomical features. The right cephalic and trunk disks are omitted for clarity. The dash line represents the outline of the apical organ, gut, esophagus and underside of anterior and posterior lobes in sagittal mid-plane. Primary ciliary band is in blue, inner ciliary bands are in purple, ciliary ridges are brown, axils are yellow. **B** A 4-day-old pilidium of *Maculaura alaskensis* injected as a zygote with mRNA encoding GFP-UtrCH, which highlights actin-rich structures including cell boundaries. This is the stage at which the marked larvae were scored. To illustrate major epidermal cell types, one or more cells of each type are color-coded: tile cells (green), foam cells (lavender), axillary cells (yellow), multiciliated cells of the primary ciliary band (teal), and uniciliated collar cells (red). Scale bar 50 μm

Figure 1 illustrates the general anatomy of the pilidium larva and the major epidermal cell types, to which we refer throughout. A typical hat-shaped pilidium has, at its apex, an apical organ bearing a blade-like tuft of long cilia, and, at the opposite pole, two lateral lappets (left and right), an anterior lobe, and a posterior lobe, which are spanned by the primary larval ciliary band (Fig. 1A). Additionally, each lappet bears an inner ciliary band on the side facing the vestibule (the space between the lappets). The inner ciliary bands beat in the opposite direction to the primary ciliary band. The vestibule continues into a funnel-shaped esophagus, which bears two bilaterally symmetrical ciliary ridges at its posterior wall. Although the entire outer and inner surface of the pilidium larva (except for the axils) is densely ciliated, the ciliary bands stand out because they are composed of cells with distinct shape and longer, more actively beating cilia. The esophagus leads into a blind round stomach. The diagram depicts a pilidium at the trunk-disks stage, at which point two pairs of imaginal disks are present (cephalic and trunk), to illustrate the spatial relationship between the axils—the pilidial growth zones, located at the points of inflection between the lobes and lappets—and the imaginal disks. Each axil is bipartite with an outer episphere population apposed to an inner hyposphere population [14]. Cephalic disks invaginate from the outer anterior axils. Subsequently, trunk disks invaginate from the underside of the posterior lobe (probably the inner posterior axils). The fates of labeled domains were scored at an earlier developmental stage—before the formation of the imaginal disks.

Several distinct types of epidermal cells can be distinguished in a typical pilidium. The majority of the episphere (the outer surface, apical of the primary ciliary band) and hyposphere (the inner surface, vegetal of the primary ciliary band) by area is composed of large squamous multiciliated cells, which we refer to as the “tile cells” (Fig. 1B). Interspersed between the tiles of the episphere are “foam cells”—highly branched, appearing as if composed of lots of little bubbles. It is not clear whether the foam cells are ciliated, and, if so, how many cilia they bear. A cluster of small monociliated cells is located in each of the four axils [40]. The primary ciliary band is composed of numerous small and narrow multiciliated cells, among which one can differentiate a small number of regularly spaced monociliated sensory cells, each bearing a tall collar of microvilli—the so-called collar cells [3]. The primary ciliary band is a multitiered structure, which consists of the central tier bearing long rapidly beating cilia, accessory tiers of densely ciliated cells on either side, and monociliated collar cells interspersed between the cells of the central tier.

### Primary trochoblasts (1q^2^) contribute many cells to the primary ciliary band and outer axils

**Fig. 2:**
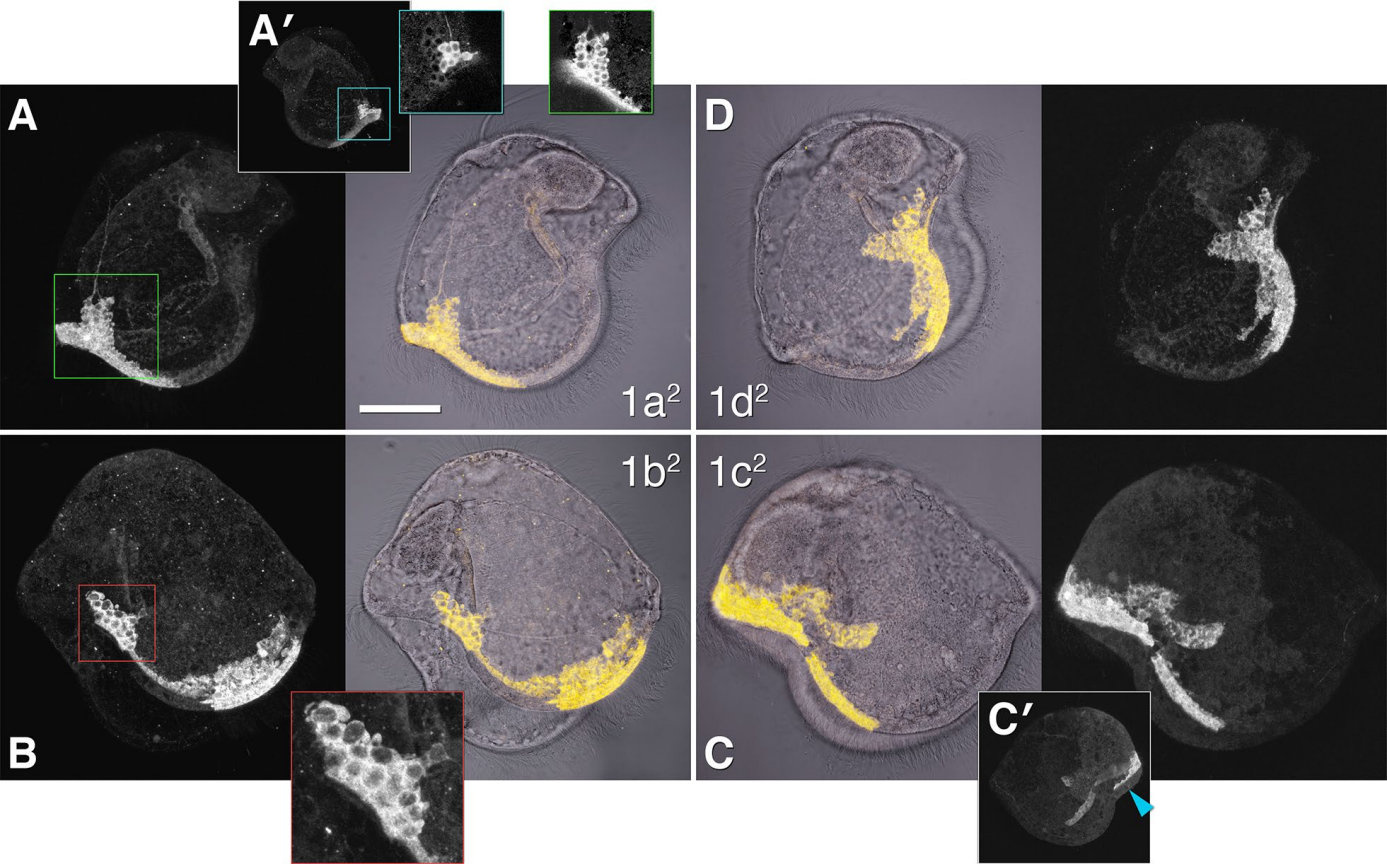
Fate of the 1q^2^ cells in the pilidium. The four middle panels show labeled domains (yellow) overlaying transmitted-light images (gray), for orientation. Peripheral panels show corresponding labeled domains alone, for clarity. Insets show enlarged or otherwise clarified views of portions of labeled domains; the color of the inset frame corresponds to the color of the outlined region of interest on the main panel. Apical organ is up on all panels. Main panels in the top row (**A**, **D**) show pilidia imaged from the left side (anterior lobe to the left); main panels in the bottom row (**B**, **C**) show pilidia imaged from the right side (anterior lobe to the right). **A** Contribution of the 1a^2^ cell: primary ciliary band, anterior left axil, a nerve. **A**′ The same larva (0.5× scale) as **A** imaged from the right side to show contribution of 1a^2^ to the anterior right axil. Insets to **A** and **A**′ (green and teal, respectively) show 1.8-μm-thick projections (same scale as **A**). Intensity values in the insets are log-transformed to make dim background visible. This highlights nuclei of axillary cells in the labeled domain because the 70 kD dextran used for labeling is excluded from nuclei. Whereas 1a^2^ makes the entire left axil, it only accounts for ~ 1/3 of cells in the right axil. **B** Contribution of the 1b^2^ cell: primary ciliary band, posterior right axil. Red inset shows an enlarged (2.5×) view of the axil. **C** Contribution of the 1c^2^ cell: primary ciliary band, episphere. **c**′ The same larva (0.5× scale) as **C** imaged from the left side to show contribution of 1c^2^ to the episphere of posterior left quadrant (arrowhead). **D** Contribution of the 1d^2^ cell: primary ciliary band, posterior left axil, episphere. Scale bar 50 μm

In the pilidiophoran *M. alaskensis*, the 1q^2^ cells, which in other spiralians cease dividing after one or two divisions and form the main portion of the prototroch, continue proliferating. Their progeny includes dozens of small cells that contribute to the primary larval ciliary band, pilidial episphere (a few tile cells), as well as the axils—the larval growth zones (Fig. 2), but the specific contributions vary between the quadrants. For example, 1a^2^ gives rise to the anterior left portion of the primary ciliary band (anterior lobe and left lappet), the entire anterior left axil (Fig. 2A), and a portion of the anterior right axil (Fig. 2A′). Uniquely among the 1q^2^, 1a^2^ does not contribute any tile cells. The 1b^2^ cell contributes to the anterior right portion of the primary ciliary band (right lappet), an adjoining portion of the episphere in the right lappet, and, somewhat surprisingly, to the posterior right axil (Fig. 2B). Its domain stretches from the vicinity of the anterior right axil (to which it does not contribute) to the posterior right axil, which it accounts for the entirety of. The progeny of 1c^2^ cell form the posterior right portion of the primary ciliary band (posterior lobe and right lappet) and a few adjoining tile cells in the episphere. Uniquely among the 1q^2^, 1c^2^ does not appear to contribute to any axil, even though the posterior right axil is in the middle of its domain (Fig. 2C, C′). Finally, 1d^2^ contributes to the posterior left portion of the primary ciliary band (left lappet), a few adjoining tile cells in the posterior left episphere, and the posterior left axil (Fig. 2D). Uniquely among the 1q^2^, 1d^2^ does not intrude into the nominal domain of another quadrant (the others do: 1a^2^ to contribute a few cells to the right axil; 1b^2^ to make the posterior axil; 1c^2^, a strip of ciliated band cells abutting the left posterior axil). Although the 1q^2^ cells contribute to all four outer axils of the pilidium, and indeed make up most of the cells in the outer axils, it is quite clear that other quartets must contribute too, because a portion of the anterior right axil remains unaccounted for. Likewise, the 1q^2^ cells account for the majority but not the entire primary ciliary band.

### Accessory trochoblasts (1q^12^) contribute to the primary ciliary band and complete the outer axils

**Fig. 3:**
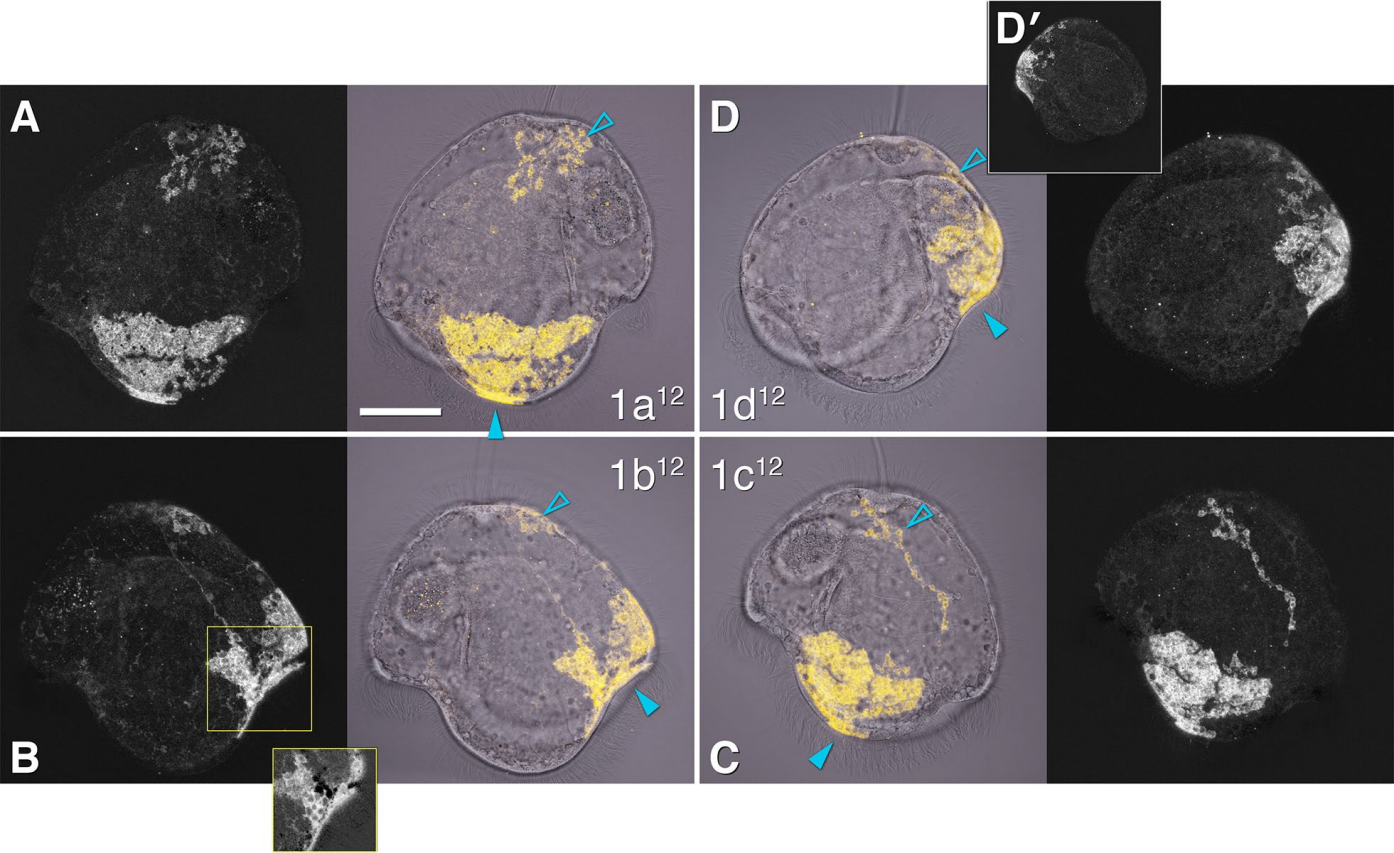
Fate of the 1q^12^ cells in the pilidium. The four middle panels show labeled domains (yellow) overlaying transmitted-light images (gray), for orientation. Peripheral panels show corresponding labeled domains alone, for clarity. Apical organ is up on all panels. Main panels in the top row (**A**, **D**) show pilidia imaged from the left side (anterior lobe to the left); main panels in the bottom row (**B**, **C**) show views from the right side (anterior lobe to the right). **A** Contribution of the 1a^12^ cell: primary ciliary band of left lappet (solid arrowhead), left-side episphere, including tile cells and foam cells (open arrowhead). **B** Contribution of the 1b^12^ cell: primary ciliary band of right lappet and anterior lobe (solid arrowhead), anterior episphere, including tile cells and foam cells (open arrowhead), and anterior right axil. Inset shows axillary region in a projection of a 1.8-μm-thick sub-stack of the same larva at the same scale, but with intensity values log-transformed to make dim background visible. The black gap in the labeled domain presumably corresponds to the contribution from 1a^2^. Note a labeled nerve. **C** Contribution of the 1c^2^ cell: primary ciliary band of right lappet (solid arrowhead), right-side episphere, including tile cells and foam cells (open arrowhead). **D** Contribution of the 1d^2^ cell: primary ciliary band (solid arrowhead) and episphere of posterior lobe, including tile cells and foam cells (open arrowhead). **D**′ The same larva (0.5×) as **D** imaged from the right side. Scale bar 50 μm

In the pilidium the 1q^12^ cells, which in other spiralians contribute to the prototroch and episphere epidermis, give rise to the tile and foam cells in the episphere on one of the lobes or lappets and a small portion of the primary ciliary band (Fig. 3). The foam cells are part of the larval episphere; they appear as long branched chains of bubbles (Fig. 1b) and are likely migratory, based on their separation from the rest of the labeled domain and inconsistencies in position from individual to individual. We guess them to be secretory cells. The tile cells are large and squamous and comprise the majority of the pilidial epidermis by area (Fig. 1b). These cells are consistent in number and position from individual to individual. The progeny of the 1a^12^ cell includes several tile cells which form the majority of the outer epidermis of the left lappet, a small portion of the primary ciliary band near the tip of the left lappet, and one or more foam cells in the left quadrant of the larval episphere, including its subapical region (Fig. 3A). The 1c^12^ domain is a near-mirror image of the 1a^12^ domain (Fig. 3C). The 1b^12^ cell gives rise to tile cells covering the episphere of the anterior lobe, a small portion of the primary ciliary band of the anterior lobe, and one or more foam cells in the anterior quadrant of the larval episphere. But, compared to its counterparts in other quadrants, it has two additional contributions—among its descendants are one or more neurons in the larval episphere, and the main portion of the anterior right axil (Fig. 3B). This axillary contribution presumably fills the vacancy left by the primary trochoblasts: 1b^2^ does not contribute to this axil at all (Fig. 2B), and 1a^2^ makes only a portion of it (compare insets to Fig. 2A′, B). Finally, 1d^12^ contributes several tile cells on the episphere of the posterior lobe, one or more foam cells in the posterior quadrant of the episphere, and a small portion of the primary ciliary band of the posterior lobe (Fig. 3D).

### Second-quartet micromeres (2q) complete the primary ciliary band and make most of the hyposphere

**Fig. 4:**
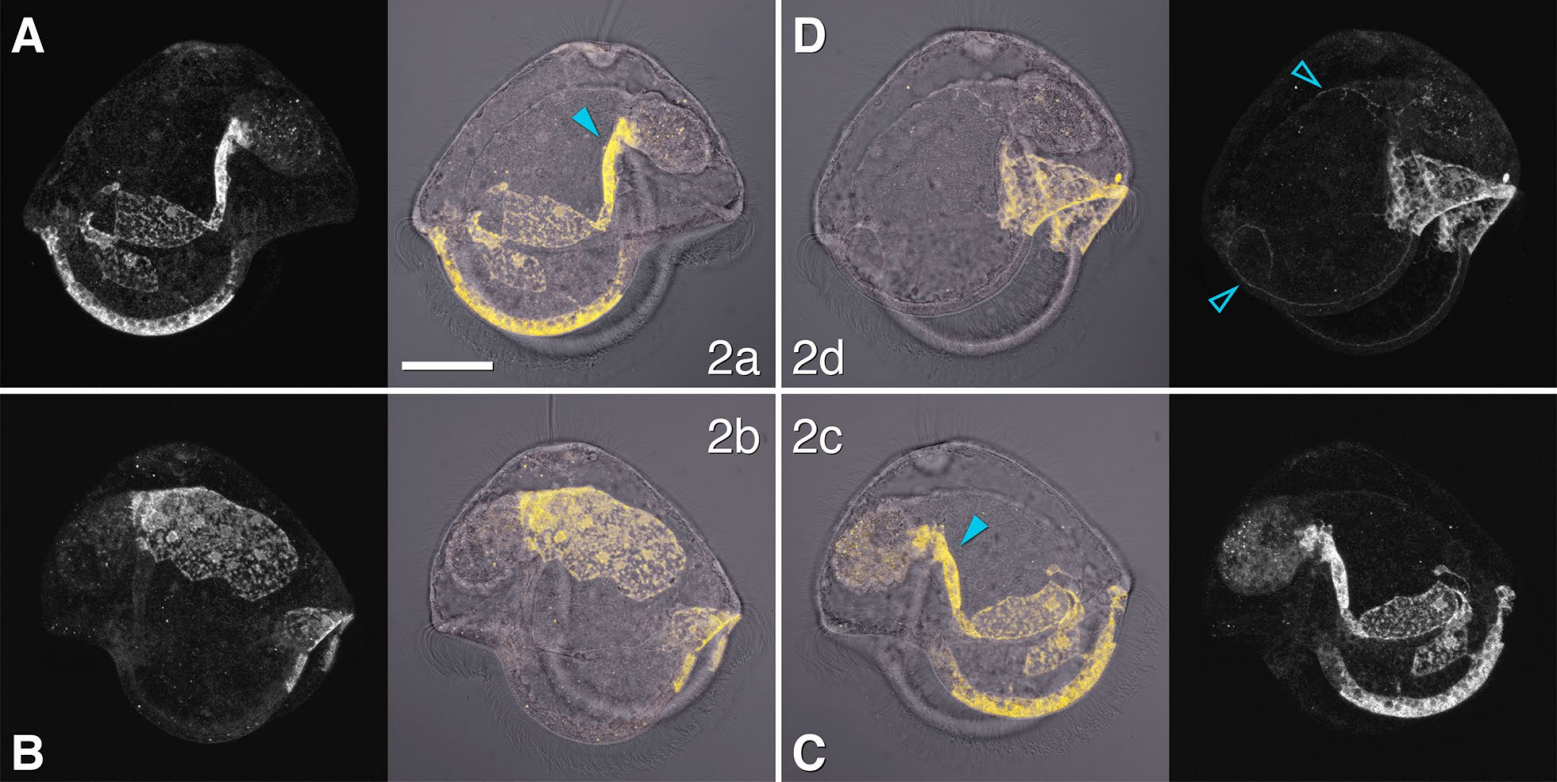
Fate of the second-quartet micromeres (2q) in the pilidium. The four middle panels show labeled domains (yellow) overlaying transmitted-light images (gray), for orientation. Peripheral panels show corresponding labeled domains alone, for clarity. Apical organ is up on all panels. Top row (**A**, **D**) shows left-side view (anterior lobe to the left); bottom row (**B**, **C**) shows right-side view (anterior lobe to the right). **A** Contribution of the 2a cell: inner ciliary band of left lappet, hyposphere tile cells of left side, left ciliary ridge (arrowhead). **B** Contribution of the 2b cell: tile cells of esophageal roof and floor of anterior lobe, primary ciliary band of anterior lobe and both lappets. **C** Contribution of the 2c cell: inner ciliary band of right lappet, hyposphere tile cells of right side, right ciliary ridge (arrowhead). **D** Contribution of the 2d cell: primary ciliary band and floor of posterior lobe, nerves (open arrowheads). Scale bar 50 μm

Second-quartet micromeres contribute a small portion of the primary ciliary band of the anterior and posterior lobes and form much of the epidermis of the pilidial hyposphere: the inner ciliary bands of the lappets, the esophageal ciliary ridges, most of the tile cells lining the esophagus, a number of tile cells lining the inner surface of the lappets, and the floor of the anterior and posterior lobes (Fig. 4). Specifically, 2a and 2c have near-mirror image contributions on the left and right sides, respectively. Each gives rise to the inner ciliary band on its respective lappet, two or more tile cells lining vestibule and esophagus, one or more neurons, one of the esophageal ciliary ridges, and a fraction of the stomach–esophagus sphincter (Fig. 4A, C). The 2b cell gives rise to the roof of the esophagus and the floor of the anterior lobe. In addition, it contributes to the inner tier of the primary ciliary band of the anterior lobe and the very anterior portions of the inner ciliary bands of the left and right lappets (Fig. 4B). This domain is roughly bilaterally symmetrical. Meanwhile, 2d also has a bilaterally symmetrical domain, which includes a portion of the primary ciliary band of the posterior lobe, and the entire floor of the posterior lobe up to the esophageal ciliary ridges (Fig. 4D). In addition to the epidermis, 2d descendants include a prominent nerve in the ciliary band of both lappets (Fig. 4D).

### Third-quartet micromeres (3q) complete the hyposphere and make larval muscles

**Fig. 5:**
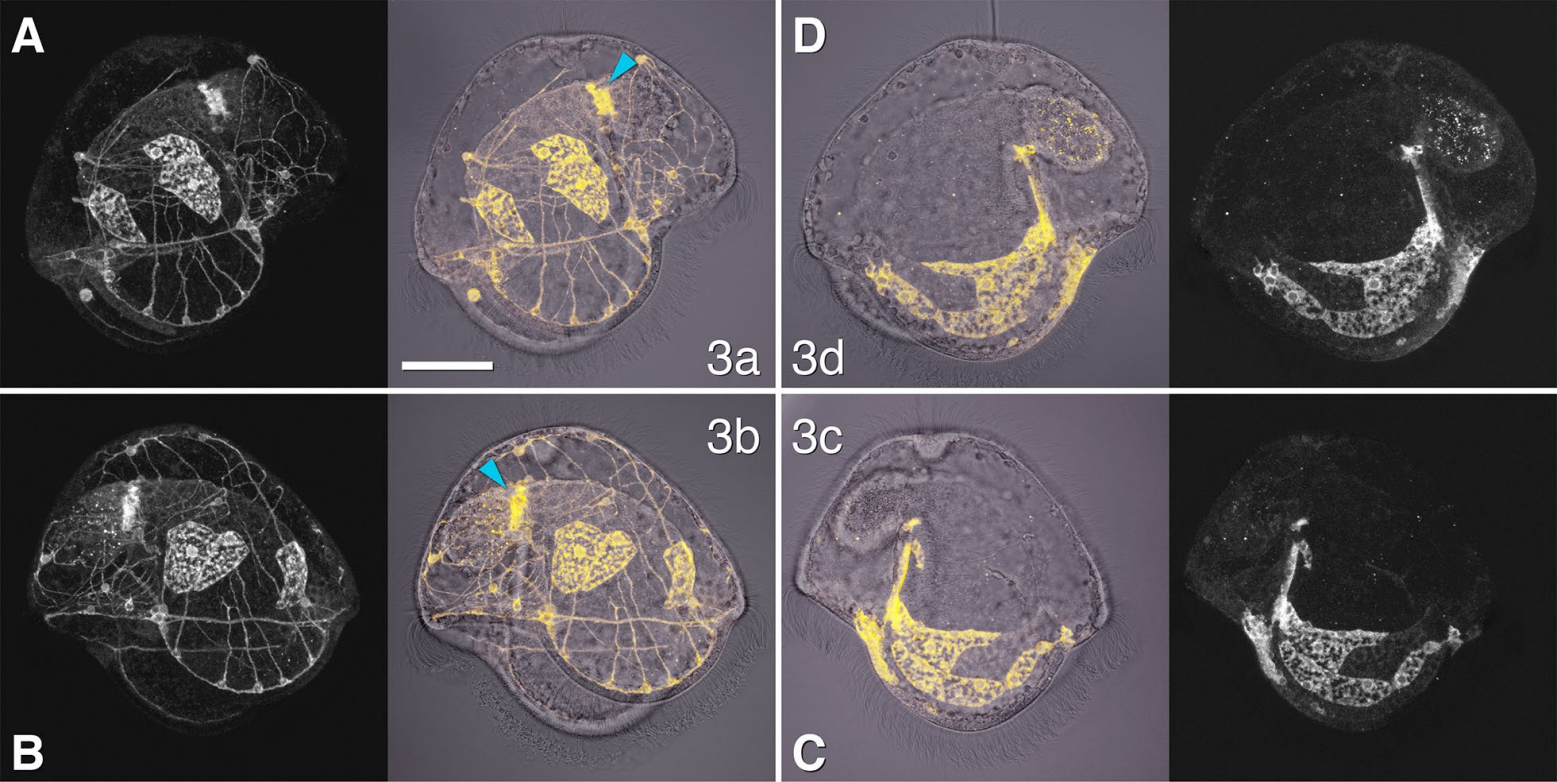
Fate of the third-quartet micromeres (3q) in the pilidium. The four middle panels show labeled domains (yellow) overlaying transmitted-light images (gray), for orientation. Peripheral panels show corresponding labeled domains alone, for clarity. Apical organ is up on all panels. Top row (**A**, **D**) shows left-side view (anterior lobe to the left); bottom row (**B**, **C**) shows right-side view (anterior lobe to the right). **A** Contribution of the 3a cell: nerves and muscles of left side, apical muscle (arrowhead), tile cells of esophageal wall of left side, esophageal sphincter (arrowhead), a nerve in the right lappet. **B** Contribution of the 3b cell: nerves and muscles of right side, tile cells of esophageal wall of right side, esophageal sphincter (arrowhead), a nerve in the left lappet, muscles of posterior lobe. **C** Contribution of the 3c cell: tile cells of inner right lappet, right and left ciliary ridge, inner ciliary band of the right lappet, cell near right outer posterior and inner anterior axils, esophageal sphincter. **D** Contribution of the 3d cell is nearly symmetrical to that of 3c, except that it does not contribute to right ciliary ridge. Scale bar 50 μm

The four cells of the third quartet form pairs (3a with 3b, 3d with 3c) with near-mirror contributions on the left and right sides, respectively (Fig. 5). The fates of 3a and 3b are quite distinct from 3c and 3d. Progeny of 3a include three tile cells in the left wall of the esophagus, larval muscles of the left side, a possible nerve spanning the margin of the right lappet, the apical muscle which connects the apical organ to esophagus, a part of esophageal sphincter, and a cluster of cell bodies in the vicinity of the inner anterior axils (both left and right) and inner posterior left axil (Fig. 5A). Because muscle or nerve fibers lead in or out of these clusters, we suspect that these are bodies of nerve or muscles cells, not axillary cells. Contributions of 3b are nearly symmetrical and include three tile cells in the right wall of the esophagus, larval muscles of the right side, a possible nerve spanning the margin of the left lappet, and part of the esophageal sphincter. The 3b cell does not, however, contribute to the apical muscle or the inner anterior right axil. On the other hand, 3b contributes to the muscles of the upper dome and posterior lobe, but 3a does not (Fig. 5B).

The 3c cell forms several tile cells of the inner side of the right lappet, a portion of the right ciliary ridge, a smaller portion of the left ciliary ridge, and a portion of the inner ciliary band of the right lappet (near posterior axil). Additionally, it makes two or three small cells in line with the cells of the primary ciliary band near the outer posterior axil, a few cells in vicinity of the right anterior inner axil, a portion of esophageal sphincter, and one or two small cells underlying the primary ciliary band roughly in the middle the right lappet (Fig. 5C). The 3d cell has a nearly symmetrical contribution on the left side, the only noticeable difference from its counterpart on the right side being that it does not contribute to the right ciliary ridge (Fig. 5D).

### Consistency of the pilidial fate map

Overall, the domains of the larval body labeled by activating each blastomere are not always compact or contiguous (e.g., 1q^12^, 2b), but they are clearly complementary to each other—i.e., they fit together like pieces of a puzzle (e.g., compare 2c and 3c). This is best visualized on a composite plate that includes all of the above-described domains aligned by quadrants (columns) and quartets (rows) (Additional file 1: Fig. S1). This complementarity is one reason why we have confidence that these domains represent normal development. Furthermore, each of these domains was observed numerous times in independent experiments (Table 1).

**Table 1:** Summary of cell-marking results

Quartet	Quadrant			
	A	B	C	D
1q^2^ Injected 1 of 4 cells^a^ 3 pools	7/8	4/8	4/7	0
1q^2^ Injected zygotes 4 pools	10/13	13/18	7/11	12/16
1q^2^ total	17/21	17/26	11/18	12/16
1q^12^ total 4 pools	17/19	20/22	22/22	11/13
2q^b^ 3 pools	24/27	20/22	29/35	2/2
2q 2 pools	34/35	36/37	38/38	22/23
2q total	58/62	56/59	77/73	24/25
3q total 2 pools	21/23	10/14	17/20	15/17

For each quartet by quadrant, the numerator corresponds to normal patterns observed—conforming to images shown in figures; denominator is the total number of scored larvae assignable to that quadrant, including those with deviant labeling patterns (e.g., missing a portion of, or having an extra part to the domain). Each type of experiment was replicated 2–4 times with separate pools of embryos

^a^Injecting one cell at the four-cell stage apparently biases D-quadrant specification: the injected cell is biased against assuming the D-quadrant fate (i.e., we got no 1d^2^ cases in any of these trials). All other experiments were done injecting dye into zygotes instead

^b^The conditions in these experiments somehow biased specification of the D quadrant, resulting in almost entire absence of 2d cases. Our best guess is that this bias comes about because the labeled cell necessarily faces the coverslip and may experience restricted respiration. In subsequent experiments, we were very careful to release the embryos promptly, and the results were no longer severely biased against 2d

**Fig. 6:**
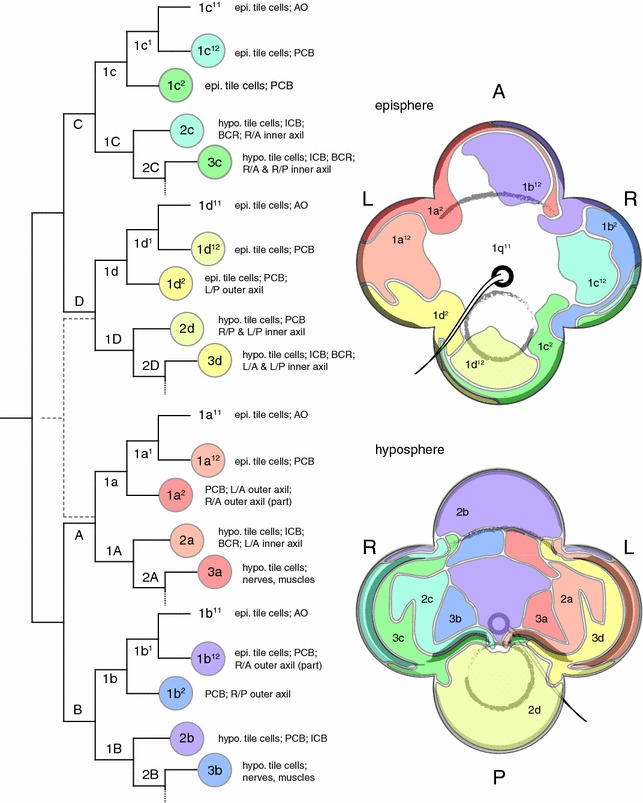
Summary of fates and spatial domains of the first, second, and third quartets in the pilidium larva. Lineage diagram on the left exhibits all cells whose fates were directly tracked in this study—1q^12^, 1q^2^, 2q, 3q—as colored nodes. We do not report data from 1q^11^, which is inferred by the difference between 1q^1^ in *C. lacteus* [25] and our account of 1q^2^ and 1q^12^. We also do not report data from 4q, but have confirmed the result reported in [25] pertains to *M. alaskensis* (data not shown). AO, apical organ; BCR, buccal ciliated ridge; epi., episphere; hypo., hyposphere; ICB, inner ciliated band; L/A, L/P, R/A, and R/P, left versus right, anterior versus posterior; PCB, primary ciliated band. Diagrams to the right depict the episphere from apical view and the hyposphere in oral view (handedness as indicated) with spatial domains for each marked cell, as inferred from Additional file 1: Fig. S1 and other data. Thickened outlines indicate portions of PCB (episphere view) or ICB and BCR (hyposphere view). For simplicity, foam cells, nerves, and muscles are omitted. Also, in the anterior and posterior lobes, contributions of 2b and 2d to the PCB are not depicted directly. In most regions, the PCB consists of several rows, including a single-row backing band with short cilia, two or three rows of heavily ciliated mainline cells, and an interband of sparsely ciliated cells interspersed with monociliated collar cells. In the right anterior and left posterior lobes, thin strips contributed by 1a^2^ and 1c^2^, respectively, appear to consist of backing band, whereas one or two rows of mainline cells are derived from 1b^12^ and 1d^12^ in the same zones. 2b and 2d also contribute to mainline cells, as well as the interband. (There is no ICB on the anterior and posterior lobes.) Note also that, as with any cartographic projection, the relative areas occupied are distorted by the imposed stretch

Nevertheless, we note that we observed some variation in patterns of labeling, particularly in larvae that appeared deformed to various degrees (e.g., undersized overall, or having an undersized lobe, a missing axil, or having an extra apical tuft or axil). Some of the atypical labeling patterns are merely deficient, i.e., a portion of the typical domain is missing. But others include deviations in which the labeled cell contributes to a structure normally produced by a different cell, e.g., in another quartet (Additional file 2: Fig. S2, Table 1). Many of these abnormalities may result from cell death after cell marking. We mention their existence because they represent non-mosaic outcomes and might therefore reveal an ability of the pilidium larva to regulate cell fates (Fig. 6).

## Discussion

Early studies of nemertean development clearly demonstrated that nemerteans possess equal spiral cleavage characterized by four embryonic quadrants and several quartets of micromeres which form through a series of synchronized divisions alternating their orientation between dextral and sinistral [e.g., 23, 24]. Hörstadius [42] inferred the fates of the four tiers of the 16-cell embryo of the pilidiophoran *Cerebratulus lacteu*s by marking isolated tiers of cells with Nile Blue and recombining them but did not label individual cells beyond the 2-cell stage. These early studies, therefore, could not answer to what extent, if any, nemertean embryos follow a spiralian developmental plan, or merely the cell division pattern. Using microinjection of a fluorescent dye into individual blastomeres, Henry and Martindale [25] determined the quadrant-specific cell fates of the four micromere quartets in *C. lacteus* and established that nemertean cell lineage is fundamentally similar to that of other spiralians. Specifically, they verified that individual blastomeres have unique and predictable fates and that these fates are similar to those in other spiralians. Key traits they documented include the orientation of the first four cells (primary embryonic quadrants) with respect to the future plane of bilateral symmetry and dual origin of mesoderm (endomesoderm from one of the 4q and ectomesoderm from 3a and 3b). They confirmed earlier findings by Hörstadius [42] that the pilidial episphere is formed by the cells of the first quartet, that the pilidial ciliary band is formed by the first and second quartets (as in other spiralians), and showed that, unlike in other spiralians, 3c and 3d cells also contribute to the ciliary band. In nearly all aspects, our analysis confirms these fundamental findings, which we principally extend by distinguishing specific contributions of 1q^2^ and 1q^12^ from the general fate of the first quartet. In addition, we can also relate fates to more recent discoveries, including the coupling of larval and imaginal growth through axils [40] and the distinction between primary ciliated band, the secondary bands along the inner lappet margin, and the esophageal ciliated ridges [3]. Finally, there are a few notable disagreements, which we discuss below.

### Radically altered fate of the “trochoblast” lineage at the heart of pilidial development

In most studied spiralians, including a palaeonemertean *Carinoma tremaphoros,* the four primary trochoblast precursors divide twice (sometimes only once), after which their daughters or granddaughters cease division and become multiciliated and, along with similarly behaving secondary and accessory trochoblasts, rearrange to form the prototroch—the primary ciliary band of the spiralian trochophore, a strictly larval structure that does not persist through metamorphosis [26, 29, 30, 39, 43–48]. This early cessation of cell division by the trochoblasts means that the cells composing the prototroch are relatively few (typically 20–40) and relatively large. In the pilidiophoran *Maculaura*, these lineages behave in a fundamentally different way: although they do form the primary ciliary band of the pilidium, they undergo many more cell divisions, before some of their progeny become multiciliated. Thus, the ciliary band of the pilidium consists of many small cells. Furthermore, some of the progeny of the “trochoblast” lineages contribute to the pilidial axils—populations of uniciliated putative stem cells which enable continuous growth of the larval epidermis (within and beyond the ciliary band) and double as the sites of origin of the imaginal disks which ultimately give rise to the juvenile [40].

The continuing expansion of larval epidermis, which results in a large inflated blastocoel supporting an extensive ciliary band used as feeding apparatus, and the formation of the imaginal disks—isolated highly proliferative rudiments that form the juvenile body—are the defining features of pilidial development. We suggest, therefore, that this particular switch in developmental programming of the trochoblast lineage—from cleavage-limited and autonomously specified to pluripotent stem cell—may, at least in part, explain the origin of pilidial development.

The constraint on cell division within the trochoblast lineage, imposed by their early adoption of the multiciliated fate, is apparently ancient: nemerteans, annelids and mollusks diverged before the Cambrian, like most extant animal phyla [19]. Because all three groups include species with typical behavior of the trochoblast lineage, we assume that this is an ancestral feature of at least the Trochozoa (which includes mollusks, annelids, and nemerteans), if not the Spiralia (Lophotrochozoa) as a whole [18, 49]. The pilidium, however, is not the only larval type that escaped this constraint. For example, among annelids, the prototroch of a lecithotrophic larva of *Capitella teleta* consists of very many (hundreds of) small cells [28]. Cell markings with microinjected fluorescent dye show that in *Capitella* the standard trochoblast lineages (1q^2^, 1q^12^, 2q with the exception of 2d) form the prototroch and that these cells continue dividing for a while before they become multiciliated and cleavage-arrested [28]. Another instance is the unusual planktotrophic mitraria larva of the annelid *Owenia,* which has a large inflated blastocoel and a long convoluted ciliary band composed of many small uniciliated cells [50], many of which continue to divide to enable the growth of the ciliary band [51]. Although the exact lineal origin of the ciliary band in the mitraria is not known, the 1q^2^ cells and adjacent blastomeres were observed to undergo multiple rounds of cell division prior to ciliation and swimming [50]. While many molluskan trochophores have a classical large-celled prototroch [reviewed in 29], in some molluskan veliger larvae the velum (whose main ciliary band is derived from the trochoblast lineage) grows considerably over the course of larval life, and thus, one can infer that there must remain some population of undifferentiated, proliferative precursors that extend or intercalate into the ciliary bands, as suggested by preliminary results of BrdU-labeling in veligers of the gastropod *Nassarius* [51]. It is possible that many if not all of continuously growing spiralian larvae with prolonged planktonic development have somehow altered the trochoblast fate typical of many direct developers, such as *Patella* [29] or *Nereis* [43].

### Unexpectedly, right cephalic imaginal disk originates from a mix of “trochoblast” lineages

Henry and Martindale [25] determined that the cephalic imaginal disks in the pilidium larva originate from the first-quartet micromeres, specifically 1a (left cephalic disk) and 1b (right cephalic disk). Although we scored our results before the cephalic imaginal disks form in *M. alaskensis*, we can extrapolate based on the knowledge of their sites of origin—the outer anterior axils [1, 40]. Our results show that the 1a^2^ cell (the vegetal daughter of 1a) accounts for the entire outer anterior left axil and thus likely forms the left cephalic disk, confirming the earlier finding by Henry and Martindale. Surprisingly, the right cephalic disk has a mixed origin from 1a^2^ and 1b^12^ (the vegetal granddaughter of the animal daughter of the 1b). Caged-FITC dextran, as used here, did not permit long-term labeling, as the combined effects of spontaneous uncaging and dilution due to growth obscured the signal around axils, imaginal disks, and other growth zones after about 1 week. However, injection of 1 of 4 cells with TRITC-Dextran or mRNA for 3xGFP-EMTB (a microtubule probe that lasts indefinitely in the pilidium; [40]) confirmed our inference: in 8- to 14-day-old larvae, when the A quadrant was injected, the entire left-side cephalic imaginal disk and a portion of the right-side disk contained label, albeit diluted by growth; when the B quadrant was injected, only a portion of the right-side disk contained label (Additional file 3: Fig. S3). Aside from the curiosity of this result—which, combined with the fate of 2d (below), implies that most of the juvenile originates from the A and D quadrants—the “linked arms” of the 1q^2^ domains also bespeak cell rearrangement relative to the cleavage pattern. These cells do not contact each other at the 16-, 32-, or 64-cell stages, while the domains of their progeny directly abut one another (except for 1d^2^ and 1a^2^, which are separated by 1a^12^).

### The primary somatoblast, 2d, forms the juvenile trunk, in keeping with its role in other spiralians

The second-quartet micromere of the dorsal quadrant, 2d, is referred to as the primary somatoblast in spiralian development; it gives rise to much of the adult body. In annelids, it forms the majority of the post-trochal ectoderm, and ultimately most of the adult trunk, and it has been shown that this cell has properties of a developmental organizer [52–56]. In mollusks, there is some variation in exact contributions, but 2d consistently gives rise to a portion (gastropods, chitons) [57–60] or most of the shell-forming mantle (bivalves) [61], and also appears to have an organizing role [62]. In the polyclad flatworm *Hoploplana,* 2d gives rise to a strip of dorso-posterior larval ectoderm [63]. In a palaeonemertean, *Carinoma tremaphoros,* 2d contributes three large cells to the vestigial prototroch and forms all of the post-trochal ectoderm and part of esophagus [26]. According to Henry and Martindale [25], 2d in the pilidium forms a portion of the pilidial ciliary band (on the posterior lobe), ectoderm of the pilidial posterior lobe and part of esophagus, plus some nerves. Our study confirms these findings, but offers a somewhat more nuanced view, in part thanks to scoring by confocal microscope rather than epifluorescence. Specifically, we show that the 2d cell forms the *entire* floor of the posterior lobe. This is notable because the trunk imaginal disks of the pilidium larva (which form most of the juvenile trunk) invaginate from the floor of the posterior lobe [1]; hence, 2d in the pilidium acts as the primary somatoblast, in accordance with its role in development of other spiralians.

### Are pilidial ciliary bands homologous to ciliary bands of other spiralian larvae?

Ciliary bands of spiralian larvae, including the prototroch—the primary locomotory organ and the defining feature of the trochophore larva [31]—feature prominently in both historical and current discussions of bilaterian evolution [e.g., 30, 64–78]. The prototroch of a typical spiralian trochophore, i.e., in many annelids and mollusks, is derived from the first-quartet (1a, 1b, 1c, 1d) and second-quartet micromeres (2a, 2b, 2c, less frequently also 2d, and in one instance—3d) [reviewed in 30]. The vestigial prototroch of the palaeonemertean *Carinoma* is derived from all four quadrants of the first- and second-quartet micromeres (1a, 1b, 1c, 1d, 2a, 2b, 2c, 2d) [26, 39]. According to Henry and Martindale [25], the ciliary band of the pilidium larva of *Cerebratulus lacteus* is composed of the descendants of all four quadrants in the first and second quartets, as well as 3c and 3d cells. They argued that the homology between the prototroch of the trochophore larva and the pilidial ciliary band is unclear: the difference in lineal origin could suggest either homoplasy or recruitment of additional lineages for ciliary band in the pilidium, or loss of the ability of those lineages to form “trochal” cilia in other spiralians. Our results clarify the origin of pilidial ciliary band. Armed with a somewhat more detailed knowledge of pilidial anatomy and function [3], we can now make a distinction between the primary ciliary band of the pilidium larva (which beats toward the vestibule, driving the primary swimming current), and the two inner ciliary bands on the lappets (which beat in the opposite direction during prey capture). Our data show that the first-quartet cells make most of the primary ciliary band; the second-quartet cells contribute to the primary ciliary band (principally 2b in the anterior lobe and 2d in the posterior lobe) and account for most of the inner ciliary bands on the lappets (2a and 2c); the third-quartet micromeres 3c and 3d do not, in fact, normally contribute to the primary ciliated band, but instead they contribute a small portion to the inner ciliary bands (among other things). Thus, with the exception of 2a and 2c contributions, the lineal origin of the primary ciliary band in the pilidium larva appears quite similar to that of other spiralians (including *Carinoma*).

Aside from the similarity of lineal origin, if one considers nemertean phylogeny [Palaeonemertea (Hoplonemertea, Pilidiophora)] [79] and the distribution of larval types across nemertean taxa, two alternative hypotheses on the origin of the pilidium’s differentially ciliated band appear plausible. The first is that the pilidial ciliary bands are not homologous to the ciliary bands of other spiralians, *as differentially ciliated bands* [10]. The larvae of palaeonemerteans and hoplonemerteans are all uniformly ciliated, and while at least one palaeonemertean larva retains a vestige of the prototroch obscured by uniform ciliation [39], even this is absent in the hoplonemerteans [80]. The only nemertean group that includes larvae with differentially ciliated bands is the Pilidiophora. In the convergent scenario, the ancestral nemertean larva would have been uniformly ciliated (while retaining a vestigial prototroch, like *Carinoma*), and the ciliary bands of the pilidium larva are derived independently from those of other spiralians. This implies one loss of differential ciliation of the trochoblast lineage in the most recent common ancestor of nemerteans, and one gain of differential ciliation in the Pilidiophora. This view is supported by the difference in function of the ciliary bands in a typical planktotrophic pilidium, such as found in *M. alaskensis* [3], and that of the opposed-band feeding mechanism described for many spiralian trochophores, including larvae of mollusks, annelids, and entoprocts [e.g., 81–89]. If this is the true history, it is intriguing that the same lineages should be co-opted to make primary larval ciliary band in the pilidium as in the prototroch of annelids and mollusks. Are “trochoblast” lineages predisposed toward forming ciliary bands because of their equatorial position in the spiralian embryo, and locomotory advantages of an equatorial ciliary band, as previously argued by Ivanova-Kazas [70], or because of some intrinsic feature of development, e.g., asymmetric inheritance of maternally derived mRNAs by cells in different quartets [90–92]?

However, an alternative scenario also appears plausible: that differential ciliation of the trochoblast-derived ciliary band was present in the most recent common ancestor of nemerteans (shared with other Trochozoans or Spiralians, more generally), and that differential ciliation was lost independently in the two other nemertean groups—once in the most recent common ancestor of the Palaeonemertea (retaining a vestigial prototroch) and once in the Hoplonemertea. Indeed, numerous transitions to lecithotrophy among Pilidiophora are accompanied by reorganization [e.g., 10] or complete loss [e.g., 93] of differentially ciliated bands [reviewed in 8].

### Evidence of cell fate regulation in pilidiophoran embryos

Spiralian development is traditionally viewed as mosaic: fates of cells are determined by lineage and are not strongly dependent on developmental context. The paramount example of this mosaic character is the isolation of the precursors of the primary trochoblasts of the limpet *Patella* [94] or *Nereis* [95], which when isolated follow the same division schedule (even orientation) and differentiation program that they would in the intact embryo. Otherwise, defects in spiralians generally yield less, not more, than would be expected on the basis of the fate map. Thus, for example, classical experiments show that ablation of the D macromere (the spiralian organizer) in the mollusk *Ilyanassa* prior to the formation of the fourth quartet results in the absence of structures derived from micromeres (e.g., eyes, shell, foot), which, therefore, are shown to depend on inductive (or at least evocative) signals [e.g., 96, 97]. More recently, Yamaguchi et al. [98] have demonstrated the ability of normally non-eye-forming micromeres in an annelid *Capitella* to regulate their fate following ablation of a normally eye-forming neighboring micromere to produce the missing eye. Similar ability for cell fate regulation has been previously shown in *Ilyanassa* [99]. This shows that some spiralian cells can make more than their normal share of structures to compensate for death of a neighboring cell. In our cell-marking experiments, atypical labeling patterns in which a portion of the standard domain is missing (of which we observed some in all experiments) likely reflect death of a subset of the progeny of the labeled cell. These were often found in larvae that appeared deformed in some way—e.g., small overall, or having undersized lappets or lobes—as if in these instances cell death had not been compensated for. This is unsurprising in a spiralian. However, some of the atypical patterns we observed imply that the pilidium has significant regulative faculty to compensate for cell death. This is the obvious interpretation for instances in which the labeled domain includes extra territory that would normally be accounted for by another cell (e.g., 3a making structures normally produced by 1a^2^: Additional file 2: Fig. S2C). Previous experiments in the pilidiophoran *Cerebratulus lacteus*, separating blastomeres at the 2- or 4-cell stage or tiers of cells at the 8- or 16-cell stage, showed limited ability to regulate [42 and works cited therein, 100]. However, our observations suggest that, in the context of apparently complete embryos, cells may be able to take on significantly different fates. If performed systematically, such defect experiments might illuminate inductive transactions that build the pilidium and then maintain it during larval growth.

## Conclusions

The nemertean pilidium has long invited comparison to other spiralian larval forms. Suggestions include a common origin with Müller’s larva of polyclads, or a highly modified trochophore (implying homology of the pilidium’s primary ciliary band to the prototroch of annelidan and molluskan trochophores), or convergent origin of ciliary bands and other larval traits. Prior fate-mapping studies in the pilidium showed that its primary ciliary band is derived largely from similar cell lineages as the prototroch of other spiralians. Our study adds a more detailed account of the behavior and contributions of the trochoblast lineages (1q^2^, 1q^12^, and 2q) in the pilidiophoran *Maculaura alaskensis.* In most other studied spiralians, including a palaeonemertean, these lineages undergo a highly restricted number of cell divisions and have a strictly larval fate. We show that although these lineages indeed make the primary ciliary band in the pilidium, their fate differs fundamentally from that in other spiralians. In particular, all of the trochoblasts undergo many rounds of cell division, and many contribute not only to the pilidial ciliary band, but also to the primary growth zones of the pilidium—the axils—which act as sources of new cells for the continuously growing ciliary band and pilidial epidermis. Furthermore, some of their descendants also give rise to the imaginal disks which ultimately form the juvenile body; thus, in stark contrast to the cleavage-limited larval fate of classic trochoblasts, some of the homologous cells in the pilidium form an indeterminate stem lineage. We suggest that these alterations of fate, for one of the most conserved lineages in spiralian development, are key to the origin of maximally indirect development as embodied in the pilidium larva. Furthermore, despite the similarity in cell origin of the pilidial primary ciliary band and the prototrochs of annelid and mollusk trochophores, we argue that these structures evolved convergently because larvae of nemertean out-groups to the Pilidiophora (the Hoplonemertea and the Palaeonemertea) lack differentially ciliated bands. Convergence is further supported by differences in structure and function of pilidial ciliary bands and those of other spiralian larvae. It remains unknown what factors determine repeated evolution of ciliary bands from similar cell lineages in spiralian larvae.

## Additional files

**Additional file 1: Fig. S1.** Summary of examined labeled domains. Fluorescence ratio confocal images, maximum intensity projected, of labeled domains organized by quadrants (columns) and quartets (rows). These are the same panels shown in Figs. 2, 3, 4 and 5. Apical organ up on all panels. Labeled domains in A and D quadrants are shown from the left side, those of B and C are shown from the right side. Scale bar 50 μm.

**Additional file 2: Fig. S2.** Examples of atypical labeling patterns. Confocal images of labeled domains (yellow) are shown overlaying transmitted-light images (gray) for orientation, as well as alone (black and white), for clarity. Apical organ up on all panels. A, B, and D show pilidia imaged from right side (anterior lobe to the right), C shows a pilidium imaged from left side (anterior lobe to the left). **(A)** An atypical 1b^2^ pattern: no label found in the outer posterior right axil (asterisk); on the other hand, outer anterior right axil (normally covered by 1a^2^ and 1b^12^) is labeled (arrowhead). **(B)** Another atypical 1b^2^ pattern: no label in the outer posterior right axil (asterisk), but there are two labeled outer anterior right axils (arrowheads). **(C)** An atypical 3a pattern: labeled domain is missing some of the esophageal tile cells, and the larval muscles; on the other hand, the domain unexpectedly includes a portion of the primary ciliary band (arrowhead), normally covered by 1a^2^. **(D)** An atypical 3c pattern: labeled domain is missing the left ciliary ridge and esophageal sphincter contributions, but unexpectedly includes a portion of the primary ciliary band (arrowhead), normally covered by the 1c^2^. Scale bar 50 μm.

**Additional file 3: Fig. S3.** Long-term tracing of quadrants confirms composite origin of right cephalic disk. (A) 8-day-old pilidium resulting from injection of one of four blastomeres with TRITC-Dextran, projection of three adjacent sections. This larva has small cephalic disks, at this stage invaginating from the axils. Approximately half is labeled by cells from what is clearly an A quadrant pattern (as shown by total projection in A’). Insets show 2.5x magnification of the boxed area, with the imaginal disk outlined in blue and the labeled subset in yellow. (B) 11-day-old pilidium resulting from injection of one of four blastomeres with mRNA encoding 3xGFP-EMTB (a microtubule marker that lasts indefinitely [40]), projection of three adjacent sections spanning the fully invaginated right cephalic disk. The total projection (B’) shows that the labeling is clearly a B quadrant, albeit with extensive dilution of injected marker in both the anterior and posterior axils due to division (as shown in [40]). Approximately half of the right cephalic disk consists of labeled cells. In these two experiments together, 17 individuals with either A or B (but not both) quadrants labeled survived to the stage at which cephalic disks were recognizable; of these, 11 had mixed labeling of the right cephalic disk; in 1, B accounted for the entire right cephalic disk; in 2 of them, a labeled B made no contribution, and in 1, A accounted for the entire right-side disk; in 2 others labeling was too faint to tell.
