# Supplementary figures and images for: The trochoblasts in the pilidium larva break an ancient spiralian constraint to enable continuous larval growth and maximally indirect development
Source: EvoDevo. 2017 Oct 25;8:19. doi: 10.1186/s13227-017-0079-5 (PMC5655816; doi:10.1186/s13227-017-0079-5)

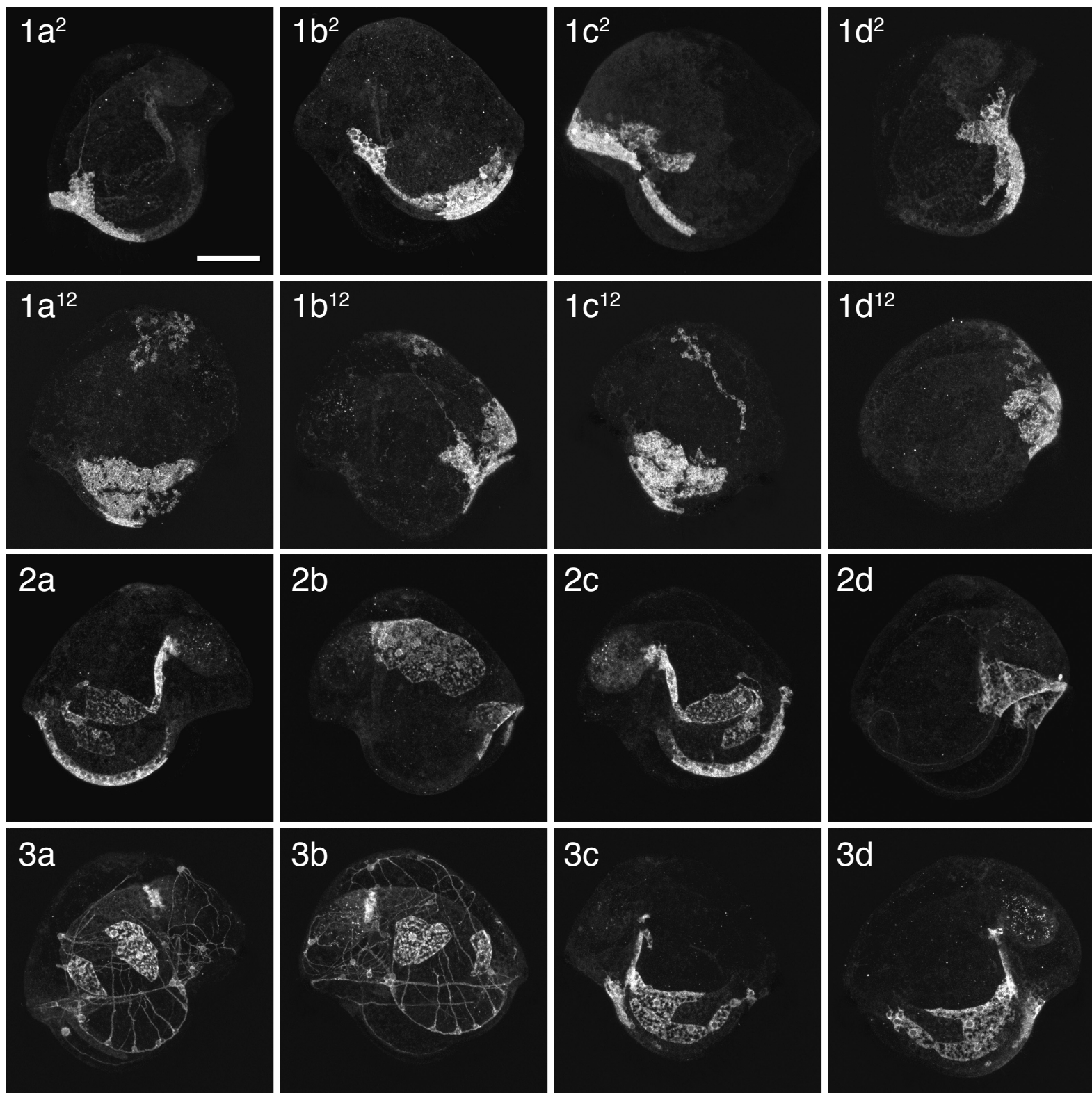

von Dassow and Maslakova Figure S1

Supplement: Supplementary file 1 — Additional file 1: Fig. S1. Summary of examined labeled domains. Fluorescence ratio confocal images, maximum intensity projected, of labeled domains organized by quadrants (columns) and quartets (rows). These are the same panels shown in Figs. 2, 3, 4 and 5. Apical organ up on all panels. Labeled domains in A and D quadrants are shown from the left side, those of B and C are shown from the right side. Scale bar 50 μm. [file 13227_2017_79_MOESM1_ESM.pdf]

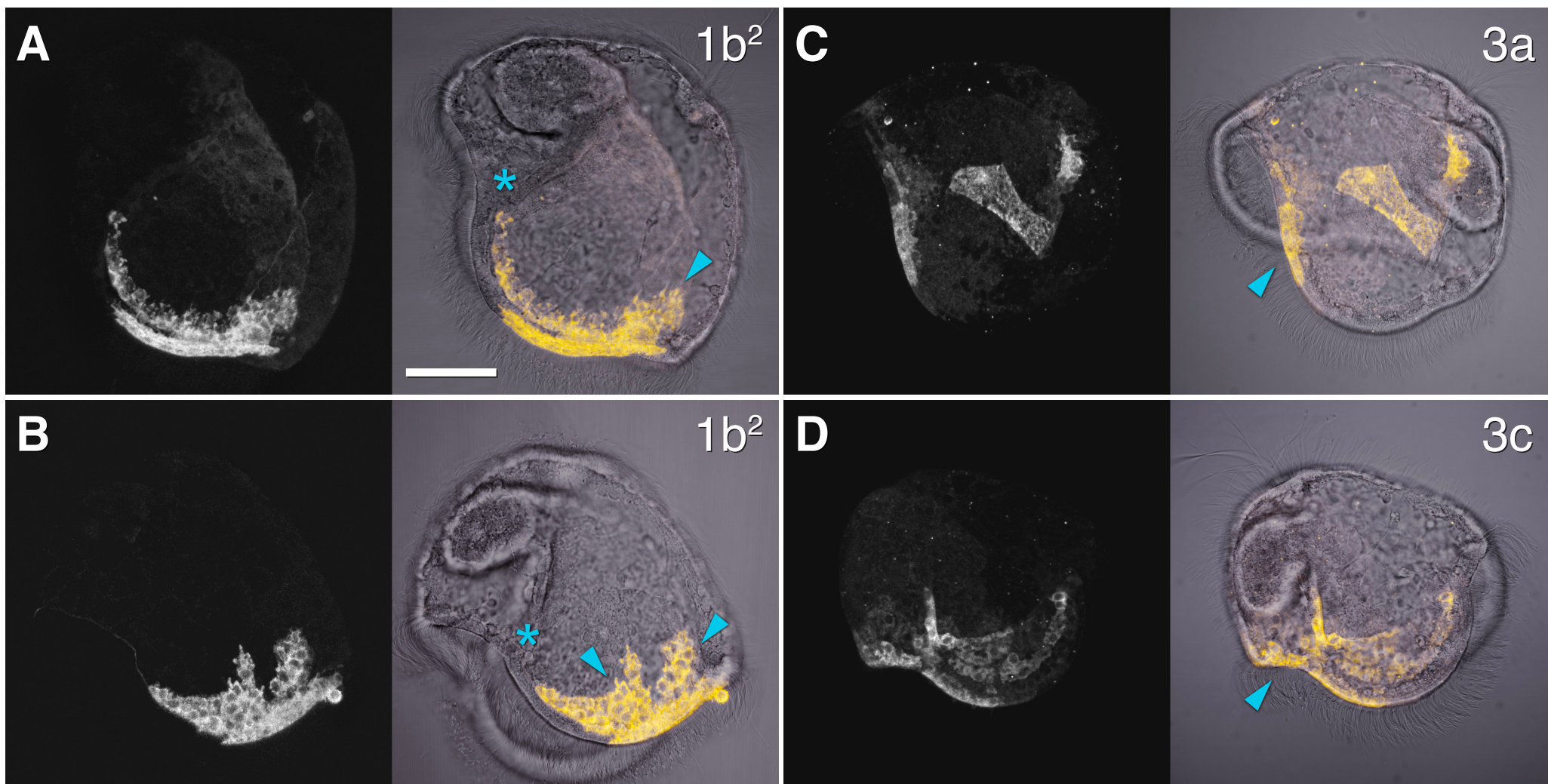

von Dassow and Maslakova Figure S2

Supplement: Supplementary file 2 — Additional file 2: Fig. S2. Examples of atypical labeling patterns. Confocal images of labeled domains (yellow) are shown overlaying transmitted-light images (gray) for orientation, as well as alone (black and white), for clarity. Apical organ up on all panels. A, B, and D show pilidia imaged from right side (anterior lobe to the right), C shows a pilidium imaged from left side (anterior lobe to the left). (A) An atypical 1b2 pattern: no label found in the outer posterior right axil (asterisk); on the other hand, outer anterior right axil (normally covered by 1a2 and 1b12) is labeled (arrowhead). (B) Another atypical 1b2 pattern: no label in the outer posterior right axil (asterisk), but there are two labeled outer anterior right axils (arrowheads). (C) An atypical 3a pattern: labeled domain is missing some of the esophageal tile cells, and the larval muscles; on the other hand, the domain unexpectedly includes a portion of the primary ciliary band (arrowhead), normally covered by 1a2. (D) An atypical 3c pattern: labeled domain is missing the left ciliary ridge and esophageal sphincter contributions, but unexpectedly includes a portion of the primary ciliary band (arrowhead), normally covered by the 1c2. Scale bar 50 μm. [file 13227_2017_79_MOESM2_ESM.pdf]

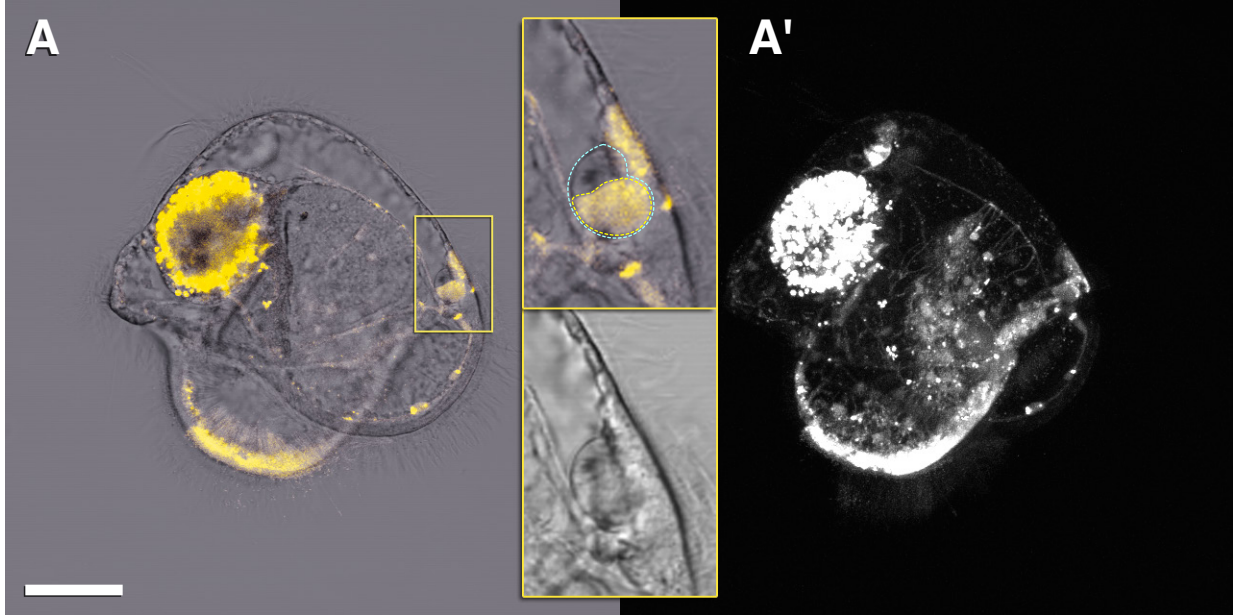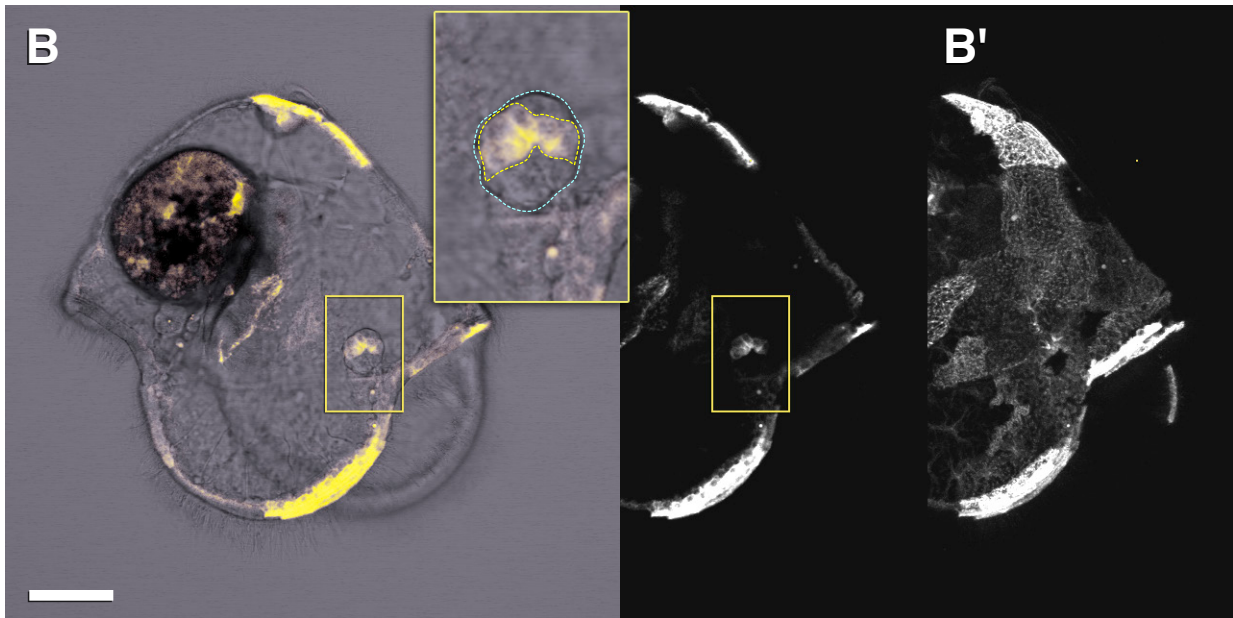

Supplement: Supplementary file 3 — Additional file 3: Fig. S3. Long-term tracing of quadrants confirms composite origin of right cephalic disk. (A) 8-day-old pilidium resulting from injection of one of four blastomeres with TRITC-Dextran, projection of three adjacent sections. This larva has small cephalic disks, at this stage invaginating from the axils. Approximately half is labeled by cells from what is clearly an A quadrant pattern (as shown by total projection in A’). Insets show 2.5x magnification of the boxed area, with the imaginal disk outlined in blue and the labeled subset in yellow. (B) 11-day-old pilidium resulting from injection of one of four blastomeres with mRNA encoding 3xGFP-EMTB (a microtubule marker that lasts indefinitely [40]), projection of three adjacent sections spanning the fully invaginated right cephalic disk. The total projection (B’) shows that the labeling is clearly a B quadrant, albeit with extensive dilution of injected marker in both the anterior and posterior axils due to division (as shown in [40]). Approximately half of the right cephalic disk consists of labeled cells. In these two experiments together, 17 individuals with either A or B (but not both) quadrants labeled survived to the stage at which cephalic disks were recognizable; of these, 11 had mixed labeling of the right cephalic disk; in 1, B accounted for the entire right cephalic disk; in 2 of them, a labeled B made no contribution, and in 1, A accounted for the entire right-side disk; in 2 others labeling was too faint to tell. [file 13227_2017_79_MOESM3_ESM.pdf]
